# Author Correction: Exploring spatial feedbacks between adaptation policies and internal migration patterns due to sea-level rise

**DOI:** 10.1038/s41467-023-39333-4

**Published:** 2023-06-14

**Authors:** Lena Reimann, Bryan Jones, Nora Bieker, Claudia Wolff, Jeroen C.J.H. Aerts, Athanasios T. Vafeidis

**Affiliations:** 1 Coastal Risks and Sea-level Rise Research Group, Department of Geography, Kiel University, Ludewig-Meyn-Straße 8, 24118 Kiel, Germany; 2 CUNY Institute for Demographic Research (CIDR), City University of New York, 135 E 22nd St, New York City, NY 10010 USA; 3 Institute for Environmental Studies (IVM), Vrije Universiteit Amsterdam, De Boelelaan 1111, 1081 HV Amsterdam, The Netherlands

**Keywords:** Socioeconomic scenarios, Climate-change adaptation, Climate-change impacts

Correction to: *Nature Communications* 10.1038/s41467-023-38278-y, published online 6 May 2023

The original version of this Article contained an error in reference 52, which incorrectly read ‘Lange, M. A. et al. in *Climate and Environmental Change in the Mediterranean Basin—Current Situation and Risks for the Future*. First Mediterranean Assessment Report (eds. Cramer, W., Guiot, J. & Marini, K.) Ch. 1 (MedECC, 2020).’ The correct version states ‘Lange, M.A. et al. Introduction. In: *Climate and Environmental Change in the Mediterranean Basin—Current Situation and Risks for the Future. First Mediterranean Assessment Report* (eds. Cramer, W., Guiot, J. & Marini, K.) Union for the Mediterranean, Plan Bleu, UNEP/MAP, Marseille, France, 41–58, 10.5281/zenodo.7100592 (2020)’. This has been corrected in both the PDF and HTML versions of the Article.

