# Supplementary material for: Exploring spatial feedbacks between adaptation policies and internal migration patterns due to sea-level rise
Source: Nat Commun. 2023 May 6;14:2630. doi: 10.1038/s41467-023-38278-y (PMC10164174; doi:10.1038/s41467-023-38278-y)
Supplement: Supplementary file 1 — Supplementary Information [file 41467_2023_38278_MOESM1_ESM.pdf]

SUPPLEMENTARY INFORMATION

Exploring spatial feedbacks between adaptation policies and internal migration patterns due to sea-level rise

Lena Reimann, Bryan Jones, Nora Bieker, Claudia Wolff, Jeroen C.J.H. Aerts, Athanasios T. Vafeidis

Supplementary Text 1 Narratives of coastal adaptation SPAs (assumptions in black font based on Ref.[1], own assumptions in blue font)

'Build with Nature'

High adaptive capacity and awareness, high national and international cooperation, and high efficiency of institutions lead to high adaptation funds being invested in proactive adaptation. With low technological barriers, adaptation strategies pursue soft, nature-based solutions such as wetland reclamation (managed realignment) and renaturation of river deltas. Furthermore, setback zones, retention areas, and managed retreat in flood-prone rural locations guarantee a decrease in exposure. In urban locations, a combination of setback zones to reduce the risk of regular coastal flooding, state-of-the-art protection measures, and policy incentives to move to cities outside flood-prone areas are pursued. Furthermore, flood warning systems are established. Also household-level adaptation is strong, proactive, and sustainable.

'Save Yourself'

Due to low adaptive capacity and awareness, limited national and international cooperation, and low efficiency in institutions, adaptation funds are extremely limited and adaptation takes place in a reactive manner, focusing on small-scale and short-term solutions, also due to high technological barriers. With no policy support, the population has to rely on household-level adaptation, which is primarily reactive and consists of simple low-cost measures. In many cases, migration away from the coast is the only adaptation measure to reduce exposure to coastal flooding.

'Hold the Line'

High adaptive capacity, high national and international cooperation, and high efficiency of institutions result in highly effective adaptation actions. Due to very high adaptation funds and low technological barriers, adaptation strategies focus on highly-engineered protective measures such as dikes, sea walls, barriers, which are well maintained and constantly upgraded as sea levels rise. Managed retreat is not actively pursued. Flood-proofing of buildings and early warning systems are put into place in locations where flooding is a recurrent problem. Household-level adaptation is practiced intensely and focusses on structural protective measures.

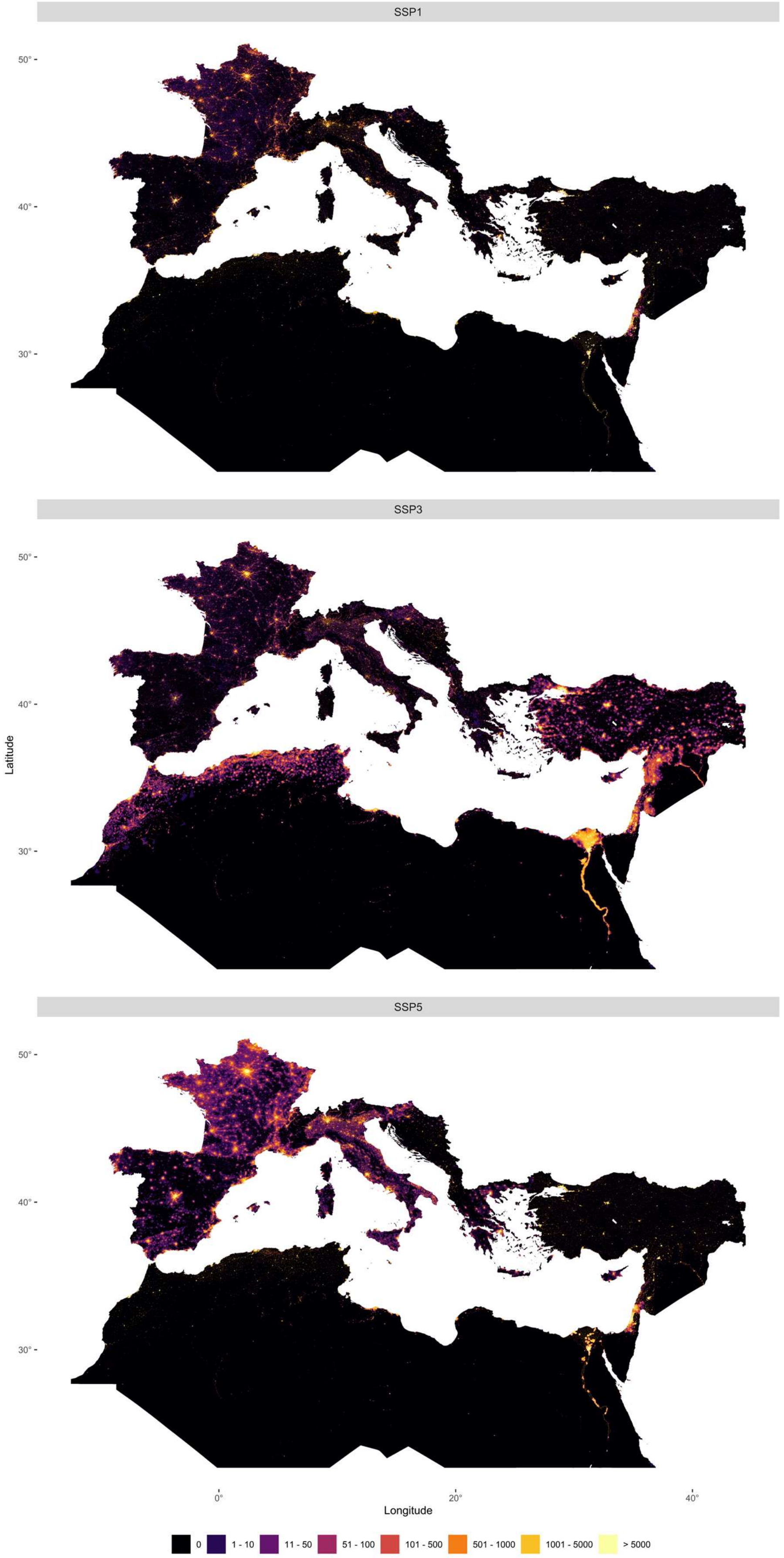

Supplementary Figure 1 Baseline 'no SLR' population projections per SSP in 2100 (based on Refs.[2,3])

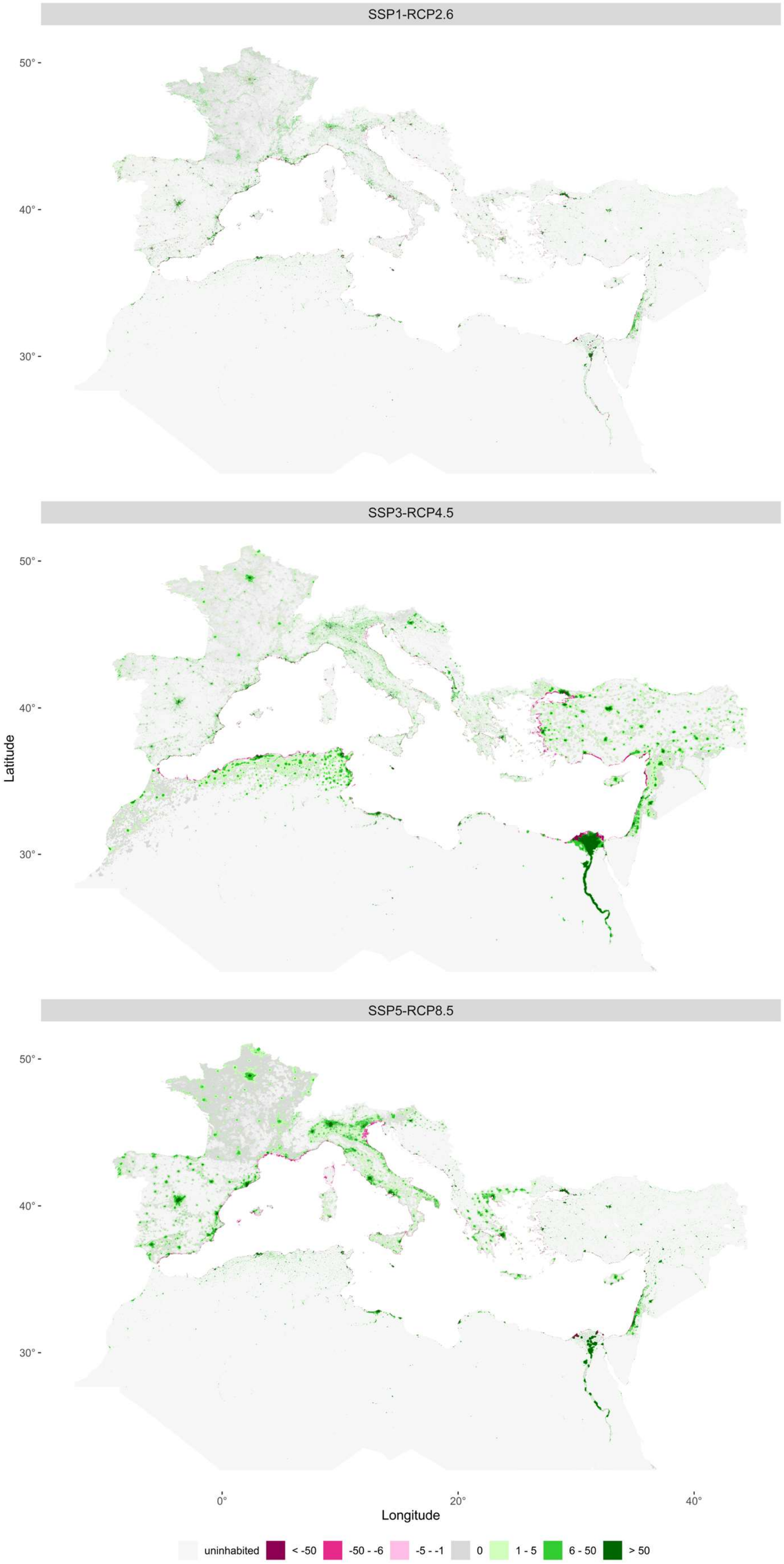

Supplementary Figure 2 Effect of SLR on spatial migration patterns in 2100 without adaptation policies. Pink colors show out-migration, green colors show in-migration compared to the no SLR baseline projections (Supplementary Figure 1)

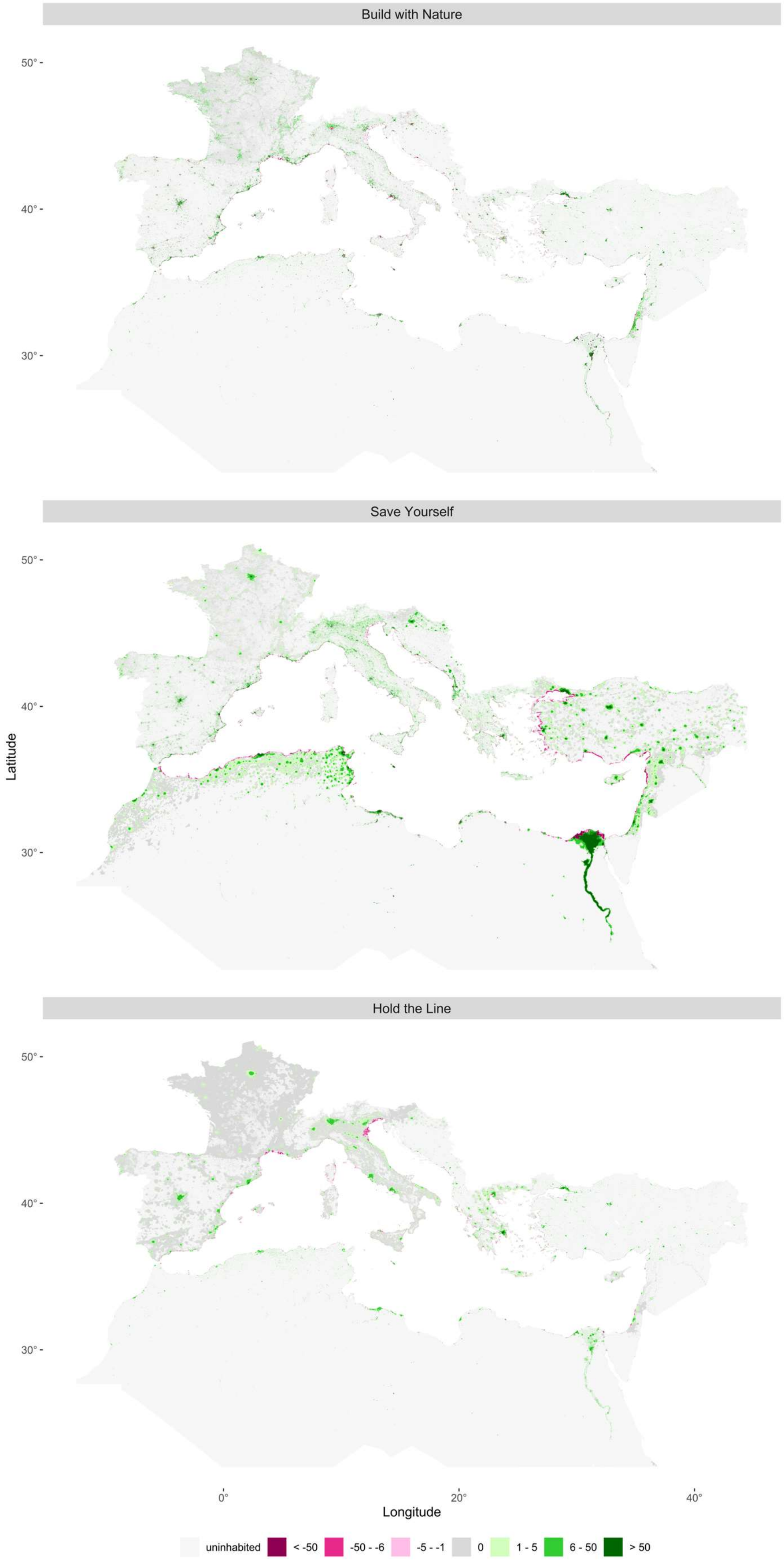

Supplementary Figure 3 Effect of SLR on spatial migration patterns in 2100 with adaptation policies. Pink colors show out-migration, green colors show in-migration compared to the no SLR baseline projections (Supplementary Figure 1)

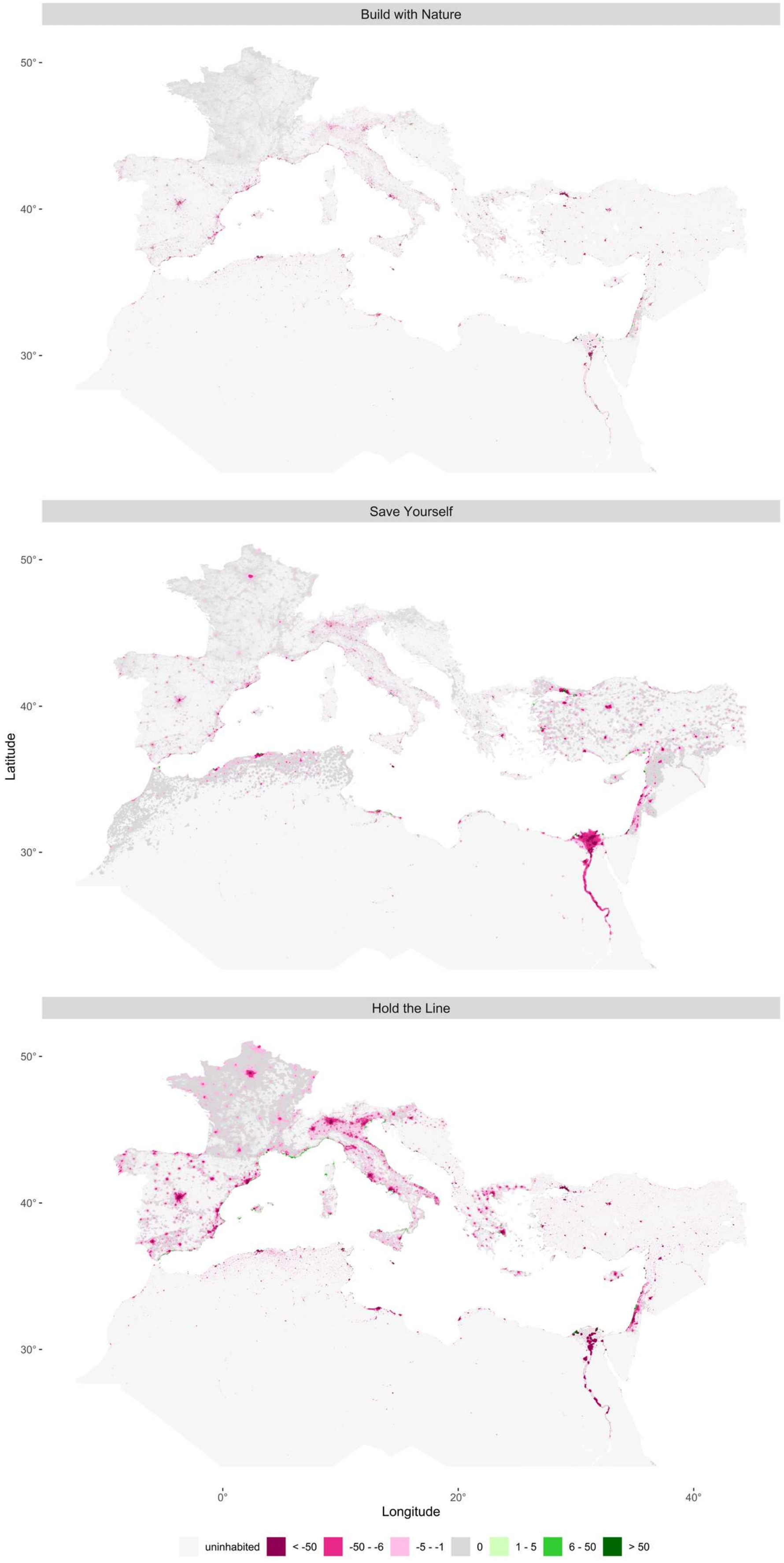

Supplementary Figure 4 Effect of adaptation policies on spatial migration patterns in 2100. Pink colors show out-migration, green colors show in-migration compared to the no adaptation policies reference projections (Supplementary Figure 2)

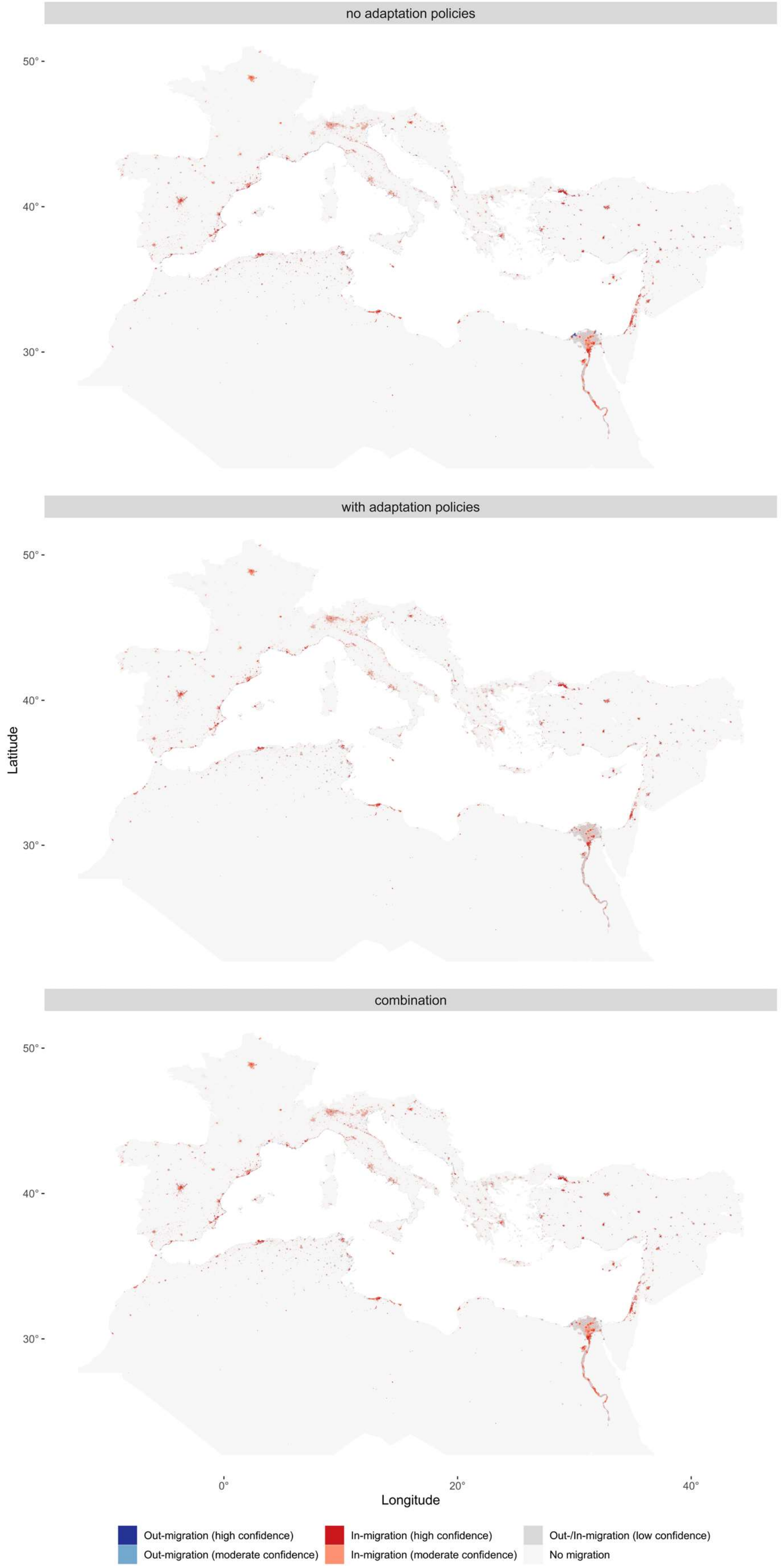

Supplementary Figure 5 Migration hotspots by 2100 for the SSP-RCP combinations without adaptation policies, with adaptation policies and across all scenarios ('combination')

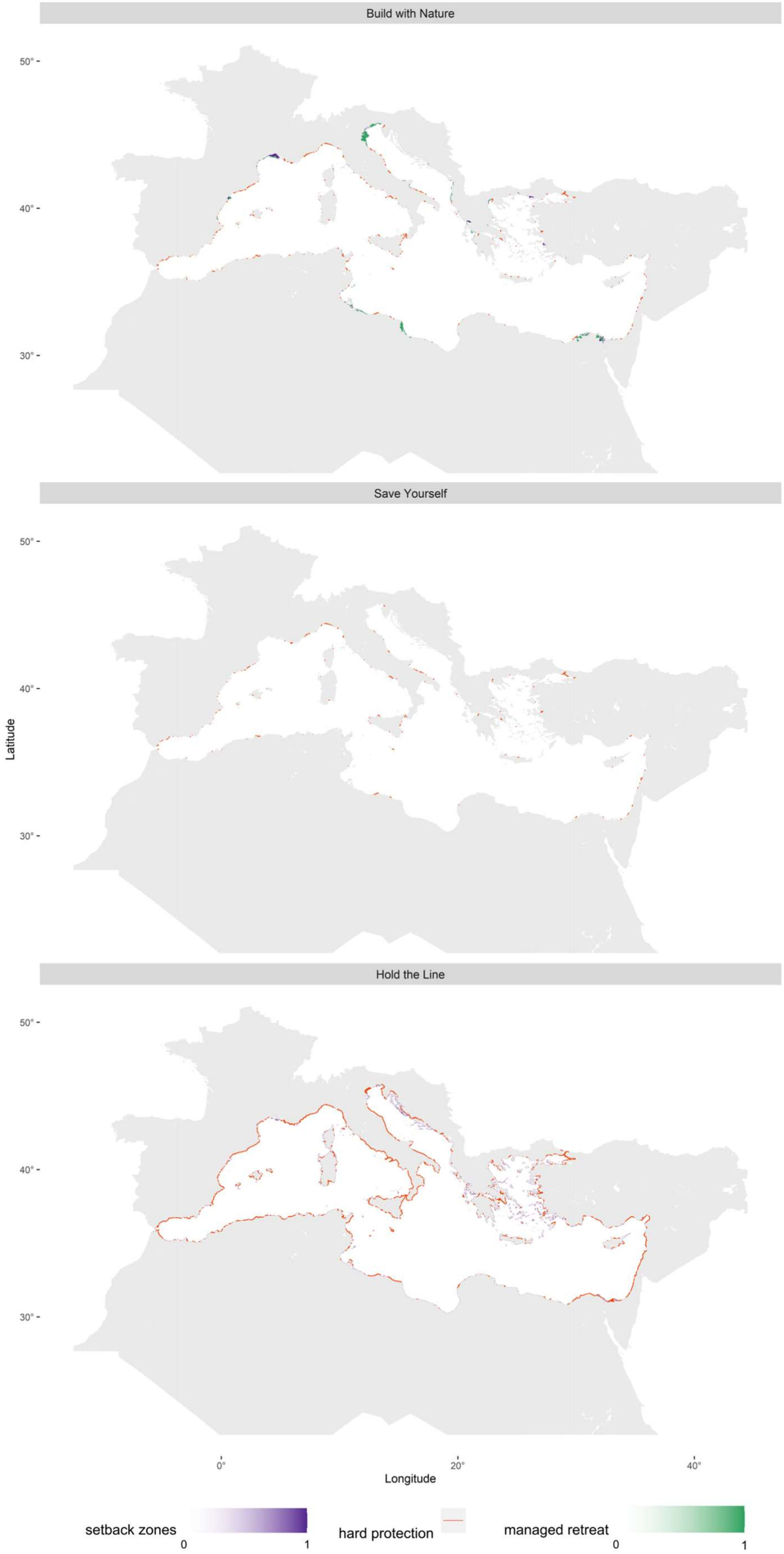

Supplementary Figure 6 Adaptation strategies per adaptation policy scenario included in this study (in 2100)

Supplementary Table 1 Countries and territories included in each geographical region (ISO 3166-1 alpha-3 country codes)

North	South & East
Andorra (AND)	Albania (ALB)
Cyprus (CYP)	Bosnia and Herzegovina (BIH)
Spain (ESP)	Algeria (DZA)
Croatia (HRV)	Egypt (EGY)
France (FRA)	Israel (ISR)
Gibraltar (GIB)	Lebanon (LBN)
Greece (GRC)	Libya (LBY)
Italy (ITA)	Montenegro (MNE)
Malta (MLT)	Morocco (MAR)
Monaco (MCO)	Palestine (PSE)
San Marino (SMR)	Syria (SYR)
Slovenia (SVN)	Tunisia (TUN)
Vatican (VAT)	Turkey (TUR)

Supplementary Table 2 Overview of coastal adaptation SPAs used in this study

	Elements	'Build with Nature' (SSP1 - RCP2.6)	'Save Yourself' (SSP3 - RCP4.5)	'Hold the Line' (SSP5 - RCP8.5)
Global SSP elements [1]	Global development path	Sustainability	Regional Rivalry	Fossil-fueled development
	Adaptation challenges	low	high	low
	GDP	moderate-high	low	very high
	International cooperation	high, effective	low	high, effective
	Policy orientation	toward sustainable development	toward security	toward development, free markets, human capital
	Institutions	effective	weak global institutions	increasingly effective
	Technological change	rapid	slow	rapid
Own adaptation policy assumptions	Adaptation policy orientation	nature-based adaptation	no adaptation policy	highly engineered solutions
	Implementation strategy	proactive	reactive	proactive
	Adaptation funds	high	low	very high
	Technological barriers	low	high	low
	Adaptation strategies	- managed retreat<br>- setback zones, incl. restoration of coastal ecosystems<br>- protection of densely populated locations	- household-level protection measures<br>- autonomous retreat<br>- protection limited to high-risk locations	- hard protection<br>- large-scale solutions

Supplementary Table 3 Criteria used for post-processing of hard protection measures produced with the DIVA modeling framework. LECZ = Low Elevation Coastal Zone

**Criteria**	**'Build with Nature' (SSP1 - RCP2.6)**	**'Save Yourself' (SSP3 - RCP4.5)**	**'Hold the Line' (SSP5 - RCP8.5)**
Settlement type	urban	urban	urban/rural
Minimum population density (pop km^{-2}) in the LECZ			
North	4,000	6,000	500
South and East	8,000	14,000	1,000

Supplementary references

1. O'Neill, B. C. *et al.* The roads ahead: Narratives for shared socioeconomic pathways describing world futures in the 21st century. *Glob. Environ. Chang.* **42**, 169–180 (2017).
2. Reimann, L., Jones, B., Nikoletopoulos, T. & Vafeidis, A. T. Accounting for internal migration in spatial population projections—a gravity-based modeling approach using the Shared Socioeconomic Pathways. *Environ. Res. Lett.* **16**, 074025 (2021).
3. Reimann, L., Jones, B., Nikoletopoulos, T. & Vafeidis, A. T. Gravity-based population projections consistent with the SSPs. *Figshare* https://doi.org/10.6084/m9.figshare.12%0A451949 (2021).
