# Supplementary material for: Exploring spatial feedbacks between adaptation policies and internal migration patterns due to sea-level rise
Source: Nat Commun. 2023 May 6;14:2630. doi: 10.1038/s41467-023-38278-y (PMC10164174; doi:10.1038/s41467-023-38278-y)
Supplement: Supplementary file 3 — Description of Additional Supplementary Files [file 41467_2023_38278_MOESM3_ESM.pdf]

Description of Additional Supplementary Files

File Name: Supplementary Data 1
Description: Number of migrants per country, scenario and settlement type from 2020-2100

File Name: Supplementary Data 2
Description: Protected coastal segments per adaptation scenario
