# Supplementary material for: Exploring spatial feedbacks between adaptation policies and internal migration patterns due to sea-level rise
Source: Nat Commun. 2023 May 6;14:2630. doi: 10.1038/s41467-023-38278-y (PMC10164174; doi:10.1038/s41467-023-38278-y)
Supplement: Supplementary file 6 — Reporting Summary [file 41467_2023_38278_MOESM6_ESM.pdf]

nature portfolio

Corresponding author(s): Lena Reimann

Last updated by author(s): Mar 20, 2023

Reporting Summary

Nature Portfolio wishes to improve the reproducibility of the work that we publish. This form provides structure for consistency and transparency in reporting. For further information on Nature Portfolio policies, see our Editorial Policies and the Editorial Policy Checklist.

Statistics

For all statistical analyses, confirm that the following items are present in the figure legend, table legend, main text, or Methods section.

n/a	Confirmed	
☒	☐	The exact sample size (*n*) for each experimental group/condition, given as a discrete number and unit of measurement
☒	☐	A statement on whether measurements were taken from distinct samples or whether the same sample was measured repeatedly
☒	☐	The statistical test(s) used AND whether they are one- or two-sided<br>*Only common tests should be described solely by name; describe more complex techniques in the Methods section.*
☒	☐	A description of all covariates tested
☒	☐	A description of any assumptions or corrections, such as tests of normality and adjustment for multiple comparisons
☒	☐	A full description of the statistical parameters including central tendency (e.g. means) or other basic estimates (e.g. regression coefficient) AND variation (e.g. standard deviation) or associated estimates of uncertainty (e.g. confidence intervals)
☒	☐	For null hypothesis testing, the test statistic (e.g. *F*, *t*, *r*) with confidence intervals, effect sizes, degrees of freedom and *P* value noted<br>*Give P values as exact values whenever suitable.*
☒	☐	For Bayesian analysis, information on the choice of priors and Markov chain Monte Carlo settings
☒	☐	For hierarchical and complex designs, identification of the appropriate level for tests and full reporting of outcomes
☒	☐	Estimates of effect sizes (e.g. Cohen's *d*, Pearson's *r*), indicating how they were calculated

Our web collection on statistics for biologists contains articles on many of the points above.

Software and code

Policy information about availability of computer code

Data collection	No specific software was used for data collection.
Data analysis	The model code for producing the spatial migration projections is available from Ref. 145 (see article) and https://github.com/lena-reimann/CONCLUDE.git.

For manuscripts utilizing custom algorithms or software that are central to the research but not yet described in published literature, software must be made available to editors and reviewers. We strongly encourage code deposition in a community repository (e.g. GitHub). See the Nature Portfolio guidelines for submitting code & software for further information.

Data

Policy information about availability of data

All manuscripts must include a data availability statement. This statement should provide the following information, where applicable:

- Accession codes, unique identifiers, or web links for publicly available datasets
- A description of any restrictions on data availability
- For clinical datasets or third party data, please ensure that the statement adheres to our policy

All input data to CONCLUDE produced in this study are based on publicly available data sources as described in the Methods and available from the corresponding references. The stretches of the coastline protected with hard protection measures per integrated scenario, generated with the DIVA modeling framework, are provided in Supplementary Data 2.

Field-specific reporting

Please select the one below that is the best fit for your research. If you are not sure, read the appropriate sections before making your selection.

☐ Life sciences ☐ Behavioural & social sciences ☒ Ecological, evolutionary & environmental sciences

For a reference copy of the document with all sections, see nature.com/documents/nr-reporting-summary-flat.pdf

Ecological, evolutionary & environmental sciences study design

All studies must disclose on these points even when the disclosure is negative.

Study description	We assess migration due to sea-level rise in a spatially explicit manner under a range of socioeconomic and sea-level rise scenarios, exploring how different adaptation policies would affect spatial and temporal migration patterns.
Research sample	We conduct this study in the Mediterranean region, using global-scale, publicly available datasets from which we extract the Mediterranean region. These datasets are primarily geographic raster data (30 arc seconds resolution), including the current (2010) and future (2020-2100) population; coastal submergence layers due to sea-level rise per time step; and different adaptation policy layers that integrate managed retreat and setback zones (based on extreme sea levels); coastal wetland restoration locations; and dike locations (Supplementary Data 2). All data and how we processed them are described in further detail in the Methods.
Sampling strategy	We did not sample any data, but used the full datasets as provided in the corresponding references (see Methods for further detail).
Data collection	The data were downloaded from the respective data sources. No further data collection took place.
Timing and spatial scale	We did not collect any data other than downloading the publicly available datasets from the respective references. From all global-scale datasets, we extracted the Mediterranean region as our study region.
Data exclusions	No data were excluded from the analysis.
Reproducibility	We have made the code for producing the migration projections available in a GitHub repository (see Code Availability Statement).
Randomization	We did not apply any randomization as the study was based on publicly available datasets. We used the full datasets provided.
Blinding	We did not blind any data as the study was based on publicly available datasets. We used the full datasets provided.

Did the study involve field work? ☐ Yes ☒ No

Reporting for specific materials, systems and methods

We require information from authors about some types of materials, experimental systems and methods used in many studies. Here, indicate whether each material, system or method listed is relevant to your study. If you are not sure if a list item applies to your research, read the appropriate section before selecting a response.

Materials & experimental systems

n/a	Involved in the study
☒	☐ Antibodies
☒	☐ Eukaryotic cell lines
☒	☐ Palaeontology and archaeology
☒	☐ Animals and other organisms
☒	☐ Human research participants
☒	☐ Clinical data
☒	☐ Dual use research of concern

Methods

n/a	Involved in the study
☒	☐ ChIP-seq
☒	☐ Flow cytometry
☒	☐ MRI-based neuroimaging
